# Novel variant in the *CNNM2* gene associated with dominant hypomagnesemia

**DOI:** 10.1371/journal.pone.0239965

**Published:** 2020-09-30

**Authors:** Alejandro García-Castaño, Leire Madariaga, Montserrat Antón-Gamero, Natalia Mejia, Jenny Ponce, Sara Gómez-Conde, Gustavo Pérez de Nanclares, Ana Belén De la Hoz, Rosa Martínez, Laura Saso, Idoia Martínez de LaPiscina, Inés Urrutia, Olaia Velasco, Aníbal Aguayo, Luis Castaño, Sonia Gaztambide

**Affiliations:** 1 Biocruces Bizkaia Health Research Institute, CIBERDEM, CIBERER, Bizkaia, Spain; 2 Paediatric Nephrology Department, Biocruces Bizkaia Health Research Institute, Hospital Universitario Cruces, CIBERDEM, CIBERER, University of the Basque Country (UPV-EHU), Bizkaia, Spain; 3 Paediatric Nephrology Unit, Hospital Universitario Reina Sofía, Córdoba, Spain; 4 Faculty of Medicine, University of Los Andes, Bogotá, Colombia; 5 Paediatric Department, Hospital Nacional Docente Madre-Niño San Bartolomé, Lima, Peru; 6 Biocruces Bizkaia Health Research Institute, Bizkaia, Spain; 7 Biocruces Bizkaia Health Research Institute, Hospital Universitario Cruces, CIBERDEM, CIBERER, Bizkaia, Spain; 8 Biocruces Bizkaia Health Research Institute, CIBERER, Bizkaia, Spain; 9 Endocrinology and Nutrition Department, Biocruces Bizkaia Health Research Institute, Hospital Universitario Cruces, CIBERDEM, CIBERER, University of the Basque Country (UPV-EHU), Bizkaia, Spain; German Cancer Research Center (DKFZ), GERMANY

## Abstract

The maintenance of magnesium (Mg2+) homeostasis is essential for human life. The Cystathionine-β-synthase (CBS)-pair domain divalent metal cation transport mediators (CNNMs) have been described to be involved in maintaining Mg2+ homeostasis. Among these CNNMs, CNNM2 is expressed in the basolateral membrane of the kidney tubules where it is involved in Mg2+ reabsorption. A total of four patients, two of them with a suspected disorder of calcium metabolism, and two patients with a clinical diagnosis of primary tubulopathy were screened for mutations by Next-Generation Sequencing (NGS). We found one novel likely pathogenic variant in the heterozygous state (c.2384C>A; p.(Ser795*)) in the *CNNM2* gene in a family with a suspected disorder of calcium metabolism. In this family, hypomagnesemia was indirectly discovered. Moreover, we observed three novel variants of uncertain significance in heterozygous state in the other three patients (c.557G>C; p.(Ser186Thr), c.778A>T; p.(Ile260Phe), and c.1003G>A; p.(Asp335Asn)). Our study shows the utility of Next-Generation Sequencing in unravelling the genetic origin of rare diseases. In clinical practice, serum Mg2+ should be determined in calcium and PTH-related disorders.

## Introduction

Magnesium (Mg2+) is an essential ion for human life that is known to play a central role in the regulation of numerous cellular functions [1]. Thus, most enzymes depend on Mg2+ as either an activator or a cofactor [2, 3]. Moreover, Mg2+ has anti-inflammatory properties and acts as calcium antagonist within cells [4]. Furthermore, Mg2+ is critical for mitochondria to carry out oxidative phosphorylation, as well as for cell proliferation, cell signaling and nucleotide binding [5]. Therefore, the maintenance of Mg2+ homeostasis is essential.

Mg2+ is the second most abundant intracellular cation [more or less 24–73 mg/dL] only surpassed by calcium. Only 1% of the body's Mg2+ is found in the blood [1.7–2.5 mg/dL] [1]. The Cystathionine-β-synthase (CBS)-pair domain divalent metal cation transport mediators (CNNMs) have been described to be involved in maintaining Mg2+ homeostasis. This family includes four proteins (CNNM1, CNNM2, CNNM3 and CNNM4) [6]. Among these, the CNNM2 protein, that was described in 2003 [7], is ubiquitously expressed, although mostly at the basolateral membrane in both the thick ascending limb of the Henle's loop, and the distal collecting tubule within the kidney where it is involved in Mg2+ reabsorption [8]. It is also expressed abundantly in brain, lung, spleen, testis, liver and heart [9]. The exact mechanism by which these proteins facilitate the passing of Mg2+ through the cell membranes has been widely discussed [10, 11]. These proteins could be themselves Mg2+ transporters [6, 10] or regulate other proteins that transport Mg2+ [11].

The CNNM2 protein (UniProtKB: Q9H8M5) structurally contains an N-terminal extracellular domain (1–250 amino acids) with a large signal peptide (64 amino acids) and one glycosylation site (asparagine residue N112) [12]; a transmembrane domain (the so-called “domain of unknown function 21” or DUF21) with four predicted transmembrane α-helices (251–431 amino acids); and a cytosolic region with two important domains: the CBS-par domain (Bateman domain) (429–578 amino acids) and the cyclic nucleotide-binding homology domain (CNBH) (621–824 amino acids) [12]. CBS-par domains dimerize in a head-to-head manner forming disc-like structures that enclose central nucleotide-binding sites for Mg2+-ATP (Adenosine triphosphate). Dimerization depends on Mg2+-ATP binding and is enhanced by phosphatases of regenerating liver (PRL) binding [13]. PRL binding is mediated by a loop from CBS-pair domain that extends into the phosphatase active site [6]. The CNBH domain does not bind cyclic nucleotides. They form dimers in solution as well. CNBH mediates CBS-par domains dimerization and is required for the Mg2+ efflux activity [14]. It has been proposed that the CNNM protein activity is regulated by conformational changes in the CBS-pair domain associated with Mg2+-ATP binding [6].

**Fig 1:**
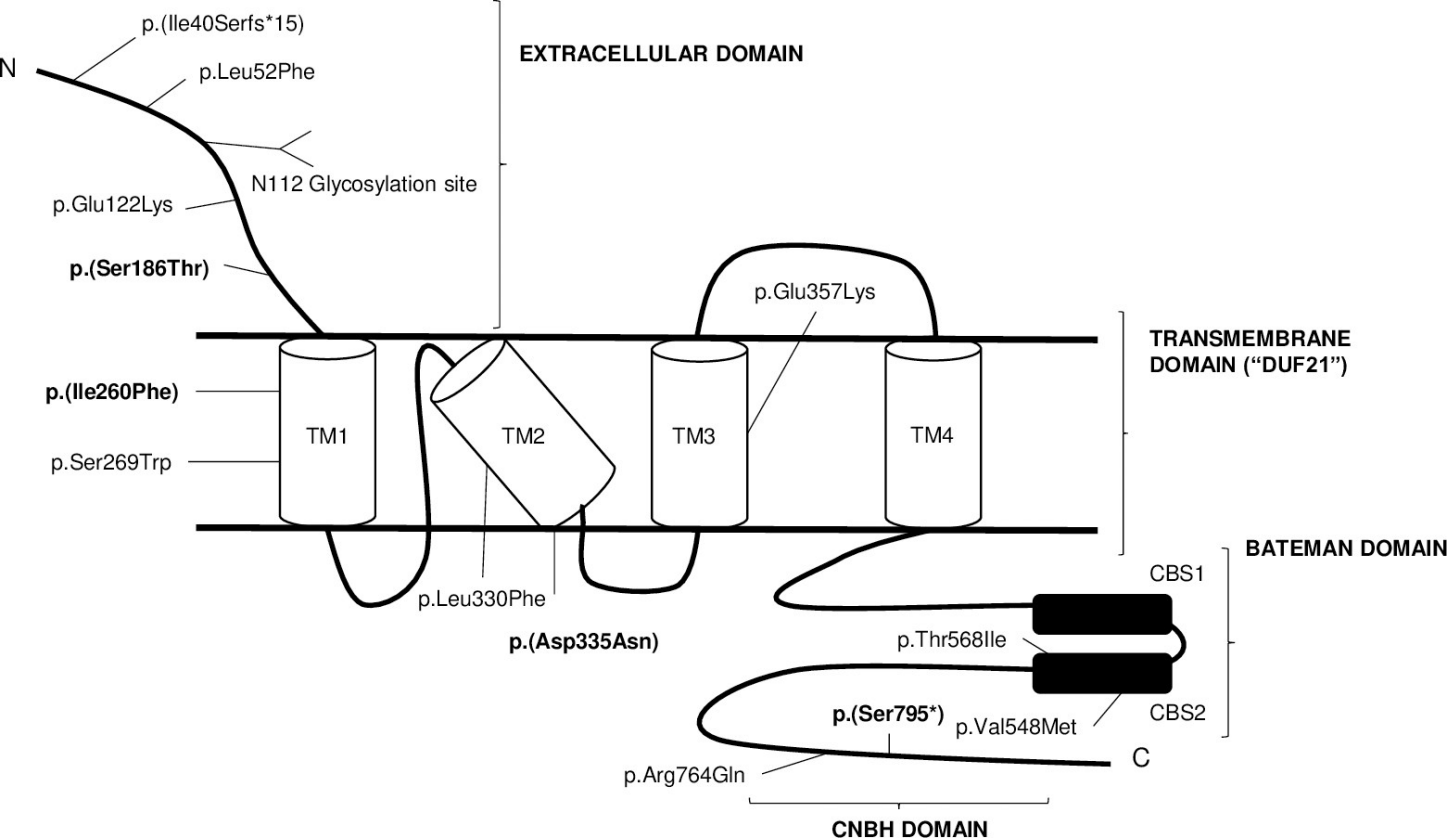
Schematic representation of CNNM2 protein at the plasma membrane, representing the position of thirteen variants. Variants marked in bold have not been reported to date. The CNNM2 protein structurally contains an N-terminal extracellular domain with one glycosylation site (asparagine residue N112); a transmembrane domain (“domain of unknown function 21” or DUF21) with four predicted transmembrane α-helices (TM1-4); and a cytosolic region with two domains: the CBS-par domain (Bateman domain) and the cyclic nucleotide-binding homology domain (CNBH).

The CNNM2 protein is encoded by the *CNNM2* gene (MIM 607803) which was mapped to chromosome 10q24.32 [15]. It has eight exons that encode a deduced 875-amino acid protein. Mutations in this gene are associated with dominant renal hypomagnesemia (MIM 613882) and with hypomagnesemia, seizures, and mental retardation (MIM 616418). So far, according to the Human Gene Mutation Database, 6 pathogenic variants in the *CNNM2* gene associated with hypomagnesemia or with hypomagnesemia, seizures, and mental retardation have been described (Table 1 and Fig 1).

**Table 1:** *CNNM2* variants previously identified in patients.

*Nucleotide change* *	*Amino acid change* *	Exon	Domain	Phenotype	Variant Class	Reference
c.117delG	p.(Ile40Serfs*15)	1	Extracellular	Hypomagnesemia	DM	Stuiver M et al [8]
c.154C>T	p.Leu52Phe	1	Extracellular	Epilepsy and autism spectrum disorder	VOUS	Long S et al [16]
c.364G>A†	p.Glu122Lys†	1	Extracellular	Mental retardation, seizures and hypomagnesemia	DM	Arjona FJ et al [17]
c.806C>G	p.Ser269Trp	1	DUF21	Mental retardation, seizures and hypomagnesemia	DM	Arjona FJ et al [17]
c.988C>T	p.Leu330Phe	1	DUF21	Mental retardation, seizures and hypomagnesemia	VOUS	Arjona FJ et al [17]
c.1069G>A	p.Glu357Lys	1	DUF21	Mental retardation, seizures and hypomagnesemia	DM	Arjona FJ et al [17]
c.1642G>A†	p.Val548Met†	2	Bateman	Hypomagnesaemia and epileptic encephalopathy	DM	Accogli A et al [18]
c.1703C>T	p.Thr568Ile	2	Bateman	Hypomagnesemia	DM	Stuiver M et al [8]
c.2291G>A	p.Arg764Gln	7	CNBH	Autism spectrum disorder Intellectual disability, seizures and epilepsy	VOUS	Kosmicki JA et al [19], Snoeijen-Schouwenaars FM et al [20]

* Numbering is according to DNA sequence (Ensembl: ENST00000369878.9)

† in homozygous; VOUS, variant of uncertain significance; DM, Disease causing mutation

In the present study, we report a novel likely pathogenic variant in the *CNNM2* gene in a Spanish family. Moreover, we report 3 novel variants of uncertain significance (VOUS) in the *CNNM2* gene.

## Materials and methods

### Ethics statement

The study was approved by the Ethics Committee for Clinical Research of Euskadi (CEIC-E). Patients and their participating relatives provided written informed consent for the genetic study.

### Population

Four patients, two of them with a suspected disorder of calcium metabolism (CA106 and SOR171), and two patients with a clinical diagnosis of primary tubulopathy (SOR79 and SOR204) were referred to our hospital for genetic testing. A total of 44 genes whose mutations are a recognized cause of primary tubulopathies were tested by a Next-Generation Sequencing (NGS) panel: patient CA106 presented with elevated calcium (Ca2+) and parathyroid hormone (PTH) levels and hypocalciuria; patient SOR171 presented with secondary hyperparathyroidism and nephrocalcinosis. On the other hand, SOR79 and SOR204 presented with hypokalemic metabolic alkalosis and were clinically diagnosed of Bartter syndrome (BS). Clinical diagnoses were made by adult and pediatric endocrinologists or nephrologists. In all cases, the molecular analysis was done in the Molecular Genetic Laboratory at Biocruces Bizkaia Health Research Institute, Barakaldo, Spain.

### Next-generation sequencing

Extraction and purification of genomic DNA from peripheral blood leukocytes were performed according to the manufacturer’s instructions (MagPurix Blood DNA Extraction Kit 200. Zinexts Life Science Corp). DNA purity and concentration were determined using Qubit 2.0 fluorometer (Thermo Fisher Scientific).

One customized gene panel with 44 genes of interest and their untranslated UTR regulatory regions was designed using the computer tool Ion AmpliSeq Designer (Ion Torrent Life Technologies). The panel is covered by 1153 amplicons, with an expected coverage of 98% and contained 44 genes whose mutations are associated with different tubulopathies (*SLC12A1*, *KCNJ1*, *BSND*, *CLCNKB*, *CLCNKA*, *CASR*, *MAGED2*, *KCNJ10*, *SLC12A3*, *SCNN1B*, *SCNN1G*, *SCN4A*, *SCNN1A*, *NR3C2*, *WNK4*, *WNK1*, *CUL3*, *KLHL3*, *ATP6V1B1*, *CA2*, *SLC4A4*, *SLC4A1*, *ATP6V0A4*, *AVPR2*, *AQP2*, *CLCN5*, *OCRL1*, *HNF1B*, *KCNA1*, *CLDN16*, *CLDN19*, *TRPM6*, *FXYD2*, *EGF*, *CNNM2*, *DMP1*, *FGF23*, *SLC34A3*, *PHEX*, *SLC34A1*, *SLC9A3R1*, *ENPP1*, *GNA11*, *AP2S1*). Libraries were prepared according to the manufacturer’s instructions (Ion AmpliSeq Library Kit 2.0). The resulting DNA libraries were ready for downstream template preparation using the Ion OneTouch™ 2 System followed by sequencing by the Ion PGM Hi-Q sequencer (Thermo Fisher Scientific). Torrent Suite^TM^ Software (Life Technologies, Foster City, CA, USA) was used for alignment to the human reference genome (hg19/GRCh37), and variant calling. Variants were filtered to include only those with a p-value < 0.001, and a Minor Allele Frequency (MAF) <1% in 1000 genomes browser, Exome Aggregation Consortium (ExAC) and ESP Exome Variant Server (ESP) and analyzed on the Ion Reporter^TM^ Software version 5.2 (Life Technologies, Foster City, CA, USA). Not appropriately covered amplicons (<20x fold) and candidate variants were assessed by Sanger sequencing.

Variants were named according to the Human Genome Variation Society guidelines, and then classified following the American College of Medical Genetics (ACMG) guidelines [21].

## Results

### Patients

Patient CA106 is a 39-year-old male who was referred for evaluation of elevated serum Ca2+ and PTH levels in several routine blood laboratory analyses and hypocalciuria. Laboratory results showed serum Ca2+ of 10.2 to 10.8 mg/dL (reference range 8.5–10.4) and high intact PTH levels (93–128 pg/mL, reference range 10–65), whereas serum phosphate (3.5 mg/dL, reference range 2.6–4.8) and 25-hydroxyvitamin D levels (39 ng/mL, reference range 9–47) were within the normal range. He was clinically diagnosed of Familial hypocalciuric hypercalcemia, therefore the *CASR* (MIM 601199), *AP2S1* (MIM 602242) and *GNA11* (MIM 139313) genes were requested for genetics analysis. Furthermore, patient CA106 had a personal history of idiopathic panhypopituitarism on substitutive hormonal treatment except growth hormone, skin sarcoma (dermatofibrosarcoma protuberans), high blood pressure, basilar artery fenestration, and he had a sprain in the wrist and back pain. We did not find alterations in the proposed genes by NGS, but we found a novel likely pathogenic variant in the heterozygous state in the *CNNM2* gene. Therefore, a new laboratory evaluation was requested confirming the previous results and the presence of moderate hypomagnesemia (1.38 mg/dL, reference range 1.7–2.5). Furthermore, hypomagnesemia was observed within his family as well (sister and mother showed hypomagnesemia, 1.32 mg/dL and 1.42 mg/dL respectively). Both had the variant carried by CA106.

Patient SOR171 is a 17-year-old male who was referred for evaluation of elevated serum PTH levels, low 25-hydroxyvitamin D levels, hypocalciuria, testicular microlithiasis and nephrocalcinosis. He was treated with 25-hydroxyvitamin D. Laboratory evaluation after 25-hydroxyvitamin D treatment showed normal serum calcium (9.2 mg/dL, reference range 8.5–10.4), high intact PTH (91.2 pg/mL, reference range 10–65), normal serum phosphate (3.7 mg/dL, reference range 2.6–4.8), serum magnesium levels at the lower limit of the normal ranges (1.7 mg/dL, reference range 1.7–2.5) and 25-hydroxyvitamin D levels of 18.5 ng/mL (reference range 9–47). A variant of uncertain significance in the *CNNM2* gene in the heterozygosis state was identified. Later, using a NGS panel of genes associated with phosphate and calcium metabolism showed a described variant of uncertain significance (c. 1039G>A; p. Val347Ile) in the heterozygosis state in the *VDR* gene (mutations in the *VDR* gene are associated with vitamin D-resistant rickets type IIA, OMIM *601769).

Furthermore, we identified variants of uncertain significance in the *CNNM2* gene in two patients previously diagnosed of Bartter syndrome. This syndrome is a heterogenic autosomal recessive disorder of the salt reabsorption at the thick ascending limb of Henle´s loop. BS is characterized by hypokalemia, metabolic alkalosis, hyperaldosteronism with normal or low blood pressure, renal salt loss and hyperplasia of juxtaglomerular apparatus [22]. In brief, patient SOR79 is a 3-month-old female with polyuria, polydipsia, vomiting, constipation, salt craving, dehydration, and failure to thrive. A history of hydramnios was also recorded. Characteristically, she had hypokalemia (serum K+ 3 mEq/l), hypochloremia (serum Cl- 86 mEq/l), metabolic alkalosis (serum pH 7.46, bicarbonate 29 mEq/l), and nephrocalcinosis. Moreover, increased plasma renin activity (500 ng/ml/h), and aldosterone levels (2008 pg/ml) were observed. At the time of the study, she had elevated serum magnesium levels (2.7 mg/dL, reference range 1.7–2.5). We identified two likely pathogenic nonsense variants in compound heterozygous state (p.[(Gln75*)];[Arg761*]) in the *SLC12A1* gene (Na-K-2Cl cotransporter NKCC2, pathogenic mutations cause type I BS (OMIM #601678)) confirming a clinical diagnosed of neonatal Bartter syndrome type 1. The second patient, SOR204, is a premature who was referred for evaluation of vomiting and hypotonia. Characteristically, he had hypokalemia (serum K+ 2.3–2.8 mEq/l), hypochloremia (serum Cl- 96–78 mEq/l), metabolic alkalosis (serum pH 7.52–7.59, bicarbonate 27.9–35.9 mEq/l), and nephrocalcinosis. At the time of the study, he had low serum magnesium levels (1.51 mg/dL, reference range 1.7–2.5). Moreover, patient SOR204 had Down syndrome and showed a delay in psychomotor development. In this patient two likely pathogenic variants in compound heterozygous state (p.[(?)];[(Glu490Lys)]) in the *CLCNKB* gene (Chloride Channel Protein ClC-Kb, pathogenic mutations cause type III BS, OMIM #607364) were identified confirming a clinical diagnosed of Bartter syndrome type 3.

Regarding molecular diagnosis, we observed 4 novel variants in the *CNNM2* gene. One likely pathogenic variant (c.2384C>A; p.(Ser795*)) in patient CA106 and three variants of uncertain significance (c.557G>C; p.(Ser186Thr) in patient SOR79, c.778A>T; p.(Ile260Phe) in patient SOR204, and c.1003G>A; p.(Asp335Asn) in patient SOR171), all in the heterozygous state (Table 2 and Fig 1). The nonsense variant p.(Ser795*) is located in the CNBH domain, and it was considered disease-causative since this change in the *CNNM2* gene disrupts the reading frame and presumably leads to a truncated protein lacking the COOH-terminus, thus hypothetically generating a faulty *CNNM2* protein unable to perform its function (Table 2).

**Table 2:** Novel *CNNM2* variants and their *in silico* pathogenicity prediction identified in this study.

Family	Gender (Female/Male)	*Nucleotide change* †	*Amino acid change* †	Exon	Domain	Polyphen2*	Mutation Taster||	Varsome [23]
SOR0079	F	c.557G>C	p.(Ser186Thr)	1	Extracellular	0.801 (Possibly Damaging)	0.9 (disease causing)	Uncertain Significance
SOR0204	M	c.778A>T	p.(Ile260Phe)	1	DUF21	0.408 (Benign)	0.9 (disease causing)	Uncertain Significance
SOR0171	M	c.1003G>A	p.(Asp335Asn)	1	DUF21	0.229 (Benign)	0.9 (disease causing)	Uncertain Significance
CA0106	M	c.2384C>A	p.(Ser795*)	7	CNBH	-	1 (disease causing)	Likely Pathogenic

† Numbering is according to DNA sequence (Ensembl: ENST00000369878.9), in heterozygous.

*Score [HumDiv range: benign 0- probably damaging 1]

|| Probability [range: 0–1].

The missenses p.(Ser186Thr) (in the extracellular domain), p.(Ile260Phe) and p.(Asp335Asn) located in the DUF21 domain were considered of the uncertain significance (Table 2).

In addition, genetic testing was done to determine whether asymptomatic family members had inherited the same variant as the index cases. Two members (mother and sister of patient CA106) had the variant p.(Ser795*) in heterozygous state and both presented with hypomagnesemia, therefore, they were clinically diagnosed of dominant hypomagnesemia. The variant p.(Asp335Asn) was not present in the remaining members of family SOR171, therefore we considered it as *de novo* variant. Finally, the variant p.(Ile260Phe) in patient SOR204 was inherited from the mother who was asymptomatic. We could not perform genetic analyses in family SOR79.

## Discussion

In this study we described 4 patients who had variants in the *CNNM2* gene. The complete genetic study revealed one likely pathogenic variant (p.(Ser795*)) in one patient with elevated serum Ca2+ and PTH levels, hypocalciuria and hypomagnesemia; and 3 variants not previously reported of uncertain significance in other three patients diagnosed of Bartter syndrome (p.(Ser186Thr) and p.(Ile260Phe)) and calcium metabolism disorder (p.(Asp335Asn)). In addition, these patients diagnosed of BS had disease causing mutations in other genes (*SLC12A1* and *CLCNKB*, respectively). Moreover, the patient with a calcium metabolism disorder had a variant of uncertain significance in the *VDR* gene.

In accordance with previous studies of the *CNNM2* gene [8, 17, 18], we found variants in the three CNNM2 regions; the extracellular domain, the transmembrane domain and the cytosolic domain. So far, only 6 pathogenic variants in the *CNNM2* gene have been reported. These pathogenic variants have been associated with hypomagnesemia, seizures, and mental retardation. Furthermore, other 3 variants of uncertain significance were described associated with hypomagnesemia, mental retardation, seizures, epilepsy and autism [16, 17, 19].

In our cohort, two patients (CA106 and SOR171) presented with elevated serum Ca+ and PTH levels and hypocalciuria. Initially, both were clinically suspected of a calcium metabolism disorder and the genetic analysis was performed according to this. We did not find pathogenic variants in the genes associated with Familiar hypocalciuric hypercalcemia (*CASR*, *GNA11* and *AP2S1*) by NGS. On the other hand, the genetic analysis showed a likely pathogenic variant (p.(Ser795*)) in patient CA106 and one VOUS (p.(Asp335Asn)) in patient SOR171, both variants in the heterozygous state in the *CNNM2* gene which is involved in magnesium homeostasis. Normally, Mg2+ is not requested in a routine blood test, therefore after the discovery of the variants in the *CNNM2* gene (gene associated with dominant hypomagnesemia) a new blood test was requested. The results showed that CA106 had low serum Mg2+. Genetic analysis were performed in his mother and sister, both had the variant p.(Ser795*) in the heterozygous state and presented with hypomagnesemia without other remarkable symptoms. This phenotype variability within the family has been observed previously in dominant hypomagnesemia due to variants in the *CNNM2* gene [8]. Thus, the initial diagnosis was changed to dominant hypomagnesemia. Regarding molecular diagnosis, the novel p.(Ser795*) variant produces a strongly truncated protein (missing more than 10% of its amino acids), and, as far as we know, it is the first described nonsense mutation associated with hypomagnesemia in the *CNNM2* gene. This nonsense variant is located within the CNBH domain. It was hypothesized that CNBH domain dimerization plays an important role in regulating CNNM2 activity [14]. The loss of this domain presumably results in a non-functional protein impairing Mg2+ reabsorption in the kidney. It has been described that homozygous mutations in the *CNNM2* gene cause hypomagnesemia, epileptic encephalopathy, brain malformations and mental retardation [18]. Nevertheless, a mild degree of intellectual disability has been described in other patients with variants in the *CNNM2* gene in the heterozygous state when these variants affect important protein domains [17]. However, this was not the case in patient CA106 (he worked as a computer programmer).

On the other hand, although SOR171 showed an overlapping phenotype with CA106, he had serum magnesium levels at the lower limit of the normal ranges, testicular microlithiasis, nephrocalcinosis and vitamin D deficiency. He had two variant of uncertain significance: one *de novo* variant p.(Asp335Asn) in the *CNNM2* gene and one variant in the *VDR* gene (c.1039G>A; p.Val347Ile), both in the heterozygous state. The variant p.(Asp335Asn) produces a conserved amino acid change. *In silico* mutation analyses predict the change as non-pathogenic. However, we hypothesize that variant p.(Asp335Asn) could have an effect on magnesium homeostasis. He presented with a similar phenotype than another patient previously described with hypocalciuria, elevated serum PTH levels and hypovitaminosis D, and normal serum calcium levels as well [18]. On the other hand, this other patient with a similar phenotype presented with hypomagnesemia. Importantly, our patient also had a variant of uncertain significance in the *VDR* gene in the heterozygous state. In most cases the disease associated with this *VDR* gene is transmitted in an autosomal recessive manner (both alleles must be altered) and patients present severe hypocalcemia and secondary hyperparathyroidism, with elevated serum levels of 1,25-dihydroxyvitamin D. However, some changes in the heterozygous state have also been described as disease causing [24]. Moreover, the *VDR* gene has been linked to nephrocalcinosis in a prevalence study of monogenic causes in paediatric patients with such alterations [25]. Unfortunately, one of the limits of our study was the lack of functional analysis; therefore, we are unable to determine whether these changes are responsible for the phenotype of the patient. However, we cannot rule out that the combination of the two alterations observed will produce the phenotype observed in our patient. Importantly, although a history of hypocalciuria was reported within the family, the parents only presented with the variant p.Val347Ile in the *VDR* gene.

Furthermore, our genetic analysis showed two missenses variants that we considered VOUS. Thus, patient SOR79 who had two pathogenic variants in compound heterozygous state in the *SLC12A1* confirming its clinical diagnosed of neonatal BS type 1, presented with the variant p.(Ser186Thr) in the *CNNM2* gene. This change is located in the CNNM2 extracellular domain. Patient SOR79 presented with hypermagnesemia, therefore this variant could be a benign polymorphism. On the other hand, patient SOR204 who had two variants in a compound heterozygous state in the *CLCNKB* gene confirming its clinical diagnosed of BS type 3, presented with the variant p.(Ile260Phe) in the *CNNM2* gene. This change is located in the DUF21 domain. The variant p.Ser269Trp, which is located in the same domain only 9 amino acids downstream of our variant, has been described associated to mental retardation, seizures and hypomagnesemia in the heterozygous state. This p.Ser269Trp variant decreases Mg2+ uptake and the expression is reduced compared with wild-type [17]. Though *in silico* mutation analyses predict the change as non-pathogenic, variant p.(Ile260Phe) may cause a similar defect in the protein. Moreover, we cannot exclude a mild effect on the phenotype since SOR204 presented with hypomagnesemia. However, hypomagnesemia is frequent in patients with pathogenic variants in the *CLCNKB* gene [26].

In conclusion, our study shows the utility of NGS and more extensive studies (whole exome-sequencing) in unravelling the genetic origin of rare diseases. Thus, a novel likely pathogenic variant in the *CNNM2* gene (p.(Ser795*)) has been found associated to dominant hypomagnesemia in a patient previously suspected of a calcium metabolism disorder. Moreover, three VOUS in the *CNNM2* gene have been found in our cohort. Among them, p.(Asp335Asn) and p.(Ile260Phe) variants may be implicated in patient's phenotypes. Mg2+ acts as a Ca2+ antagonist within cells [4]. Therefore, changes in the Mg2+ availability within the cell may cause alterations in the unbound Ca2+ fraction [1], which is an essential secondary messenger in many cellular functions. We underline that serum Mg2+ should be determined in calcium and PTH-related disorders in clinical practice. Understanding the functional impact of pathogenic variants in proteins implicated in the Mg2+ homeostasis is critical for guiding pharmacological research, and could facilitate individualized treatment of patients in the future.
